# Retraction Note: High levels of fatty acid-binding protein 5 excessively enhances fatty acid synthesis and proliferation of granulosa cells in polycystic ovary syndrome

**DOI:** 10.1186/s13048-025-01713-3

**Published:** 2025-06-16

**Authors:** Jingyu Liu, Jie Li, Xin Wu, Mei Zhang, Guijun Yan, Haixiang Sun, Dong Li

**Affiliations:** 1 Nanjing Drum Tower Hospital Clinical College of Nanjing Medical University, Nanjing, People’s Republic of China; 2 Center for Reproductive Medicine, Nanjing Drum Tower Hospital, Nanjing University Medical School, Nanjing, China; 3 Center for Obstetrics and Gynecology, Nanjing Drum Tower Hospital, Nanjing University Medical School, Nanjing, China; 4 State Key Laboratory of Reproductive Medicine, Nanjing Medical University, Nanjing, People’s Republic of China; 5 State Key Laboratory of Pharmaceutical Biotechnology, School of Life Sciences, Nanjing University, Nanjing, People’s Republic of China; 6 Center for Molecular Reproductive Medicine, Nanjing University, Nanjing, 210008 People’s Republic of China

**Retraction Note: Journal of Ovarian Research (2024) 17:44**

10.1186/s13048-024-01368-6

The Editors-in-Chief have retracted this article at the request of the authors. After the publication of this article, the authors reviewed their work and found that several immunofluorescence panels in Figs. 5G and 6D, and 6 F overlap with one another. Furthermore, the AKT and GADPH gel slices shown in the right row of Fig. 6A also overlap. The Editors-in-Chief no longer have confidence in the reliability of this article’s findings and conclusions.

All authors agree with this retraction.

